# Definition and reporting of lymphadenectomy and complete mesocolic excision for radical right colectomy: a systematic review

**DOI:** 10.1007/s00464-022-09548-5

**Published:** 2022-09-12

**Authors:** Giuseppe S. Sica, Danilo Vinci, Leandro Siragusa, Bruno Sensi, Andrea M. Guida, Vittoria Bellato, Álvaro García-Granero, Gianluca Pellino

**Affiliations:** 1 Minimally Invasive Unit, Department of Surgical Science, University Tor Vergata, Rome, Italy; 2 Department of Surgical Science, Policlinico Tor Vergata – University Tor Vergata, Rome, Italy; 3 Ospedale IRCCS San Raffaele, Milan, Italy; 4 Colorectal Unit, Hospital Universitario Son Espases, Palma, Spain; 5 Applied Surgical Anatomy Unit, Human Embryology and Anatomy Department, University of Valencia, Valencia, Spain; 6 Human Embryology and Anatomy Department, University of Islas Baleares, Palma, Spain; 7 Department of Advanced Medical and Surgical Sciences, Università Degli Studi Della Campania “Luigi Vanvitelli”, Naples, Italy; 8 Colorectal Surgery, Vall d’Hebron University Hospital, Barcelona, Spain

**Keywords:** Colorectal surgery, Colorectal cancer, Right colectomy, Complete mesocolic excision, D3 lymphadenectomy

## Abstract

**Background:**

Several procedures have been proposed to reduce the rates of recurrence in patients with right-sided colon cancer. Different procedures for a radical right colectomy (RRC), including extended D3 lymphadenectomy, complete mesocolic excision and central vascular ligation have been associated with survival benefits by some authors, but results are inconsistent. The aim of this study was to assess the variability in definition and reporting of RRC, which might be responsible for significant differences in outcome evaluation.

**Methods:**

PRISMA-compliant systematic literature review to identify the definitions of RRC. Primary aims were to identify surgical steps and different nomenclature for RRC. Secondary aims were description of heterogeneity and overlap among different RRC techniques.

**Results:**

Ninety-nine articles satisfied inclusion criteria. Eight surgical steps were identified and recorded as specific to RRC: Central arterial ligation was described in 100% of the included studies; preservation of mesocolic integrity in 73% and dissection along the SMV plane in 67%. Other surgical steps were inconstantly reported. Six differently named techniques for RRC have been identified. There were 35 definitions for the 6 techniques and 40% of these were used to identify more than one technique.

**Conclusions:**

The only universally adopted surgical step for RRC is central arterial ligation. There is great heterogeneity and consistent overlap among definitions of all RRC techniques.

This is likely to jeopardise the interpretation of the outcomes of studies on the topic. Consistent use of definitions and reporting of procedures are needed to obtain reliable conclusions in future trials. PROSPERO CRD42021241650.

**Supplementary Information:**

The online version contains supplementary material available at 10.1007/s00464-022-09548-5.

Stage at diagnosis represents the most important predictor of survival in patients with colonic cancer [1, 2]. Tumours located in the proximal colon have lower survival rates, but this association may be confined to distant-stage diagnoses [3]. Among other factors, the extent of lymphadenectomy and resection have been advocated as determinants of recurrence.

Lymphatic stations to be removed in patients with right-sided colon cancer are still a matter of discussion [4]. Discrepancies exist in terms of extent of lymphadenectomy in available guidelines, with Asian guidelines advocating extended lymph node removal (D3) on a routine basis in T3/T4 and selected T2 cancers, whereas this is still debated in other countries [5]. D3 lymphadenectomy involves removal of the lymphoadipose tissue covering the superior mesenteric vein (SMV) (also known as surgical trunk of Gillot) and the gastrocolic trunk of Henle (GCTH) [6, 7]. The authors have suggested a survival benefit in stage II and III colon cancer with D3 compared with conventional (D2) lymphadenectomy [8]. However, this is not consistently observed in the literature.

The technique proposed by Hohenberger in 2009, namely the Complete Mesocolic Excision (CME), introduced a further concept, the importance of preserving mesocolic integrity and achieving its complete removal [9]. The technique required sharp dissection between the right mesocolon and the retroperitoneum, taking as landmark the embryological plane resulting by the fusion fascia of Toldt and the fusion fascia of Fredet, followed by central vascular ligation (CVL) of ileocolic vessels, right colic vessels, and right branch of middle colic vessels [9]. Implementing CME, the authors were able to halve the local 5-year recurrence rate at their institution (6.5% vs 3.6% before and after CME use) [9].

Following the description of CME by Hohenberger, which can be combined with D3 lymphadenectomy, the role of extensive or more radical resection for colon cancer has generated growing interest, resulting in several studies being published with the aim of optimizing right colon resections for cancer. However, the definition of the procedures used for Radical Right Colectomy (RRC) has not been consistently used. Studies have been describing the technique used with different names, e.g. “CME”, “CVL”, “D3” and their variants. Until this question is solved, reliability of results presented and especially their comparison and generalizability remain poor [10–12]. This is relevant as any additional manoeuvre can produce unnecessary complications.

The aim of this study was to conduct a systematic review of all definitions for RRC, in order to identify potential discrepancies and areas for improvement.

## Methods

### Data sources and search

This is a systematic literature review performed in accordance with the current Preferred Reporting Items for Systematic Reviews and Meta-analyses (PRISMA 2020) guidelines for systematic reviews (Table S1) [13].

This systematic review was registered on PROSPERO under the protocol number CRD42021241650.

Searches were conducted for all English language full-text articles published until 31st October 2021. The following database sources were searched: PubMed (MEDLINE), Scopus, Cochrane Library, EMBASE, Web of Science.

The following term combination was used in each database: ((((complete mesocolon excision) OR (CME)) OR (D3)) OR (central vascular ligation)) AND (right hemicolectomy), ((((CME) OR (central vascular ligation)) OR (complete mesocolon excision) OR (D3)) AND (colon cancer)) NOT (right hemicolectomy), (((((CME) OR (central vascular ligation)) OR (complete mesocolon excision) OR (D3)) AND (colonic cancer)) NOT (right hemicolectomy)) NOT (colon cancer). These terms were created by one of the authors, with previous experience in systematic reviews (G.P.).

Furthermore, the references list of each selected article was analysed to identify additional relevant studies.

Records were screened for relevance based on their title and abstract, and successively, the full text of the remaining articles was analysed.

### Inclusion criteria and outcome definition

The type of studies eligible for inclusion were original articles (retrospective, prospective, randomised controlled trials [RCT]), systematic reviews and meta-analysis. The presence of a clear definition of the surgical technique in the methods section was considered a fundamental additional inclusion criterion.

Three authors (D.V., A.M.G. and L.S.) independently screened each record from full-text articles for eligibility and extracted the data, including quality analysis. Disagreement was resolved by discussion and consensus; if no agreement was reached, a fourth author was consulted (B.S.).

The primary aims were the identification of (a) the surgical steps for RRC, (b) the different nomenclatures adopted and (c) the number of reports and prevalence of each RRC step for a given technique. A surgical step for RRC was defined as a surgical manoeuvre mentioned by a given article as being exclusive to RRC as opposed to standard right colectomy. A nomenclature was defined as a particular name given to describe an RRC technique.

Secondary aims included the identification of definitions for each RRC nomenclature (each made up of a combination of the RRC steps previously identified) and the heterogeneity and overlaps in these definitions.

Heterogeneity was defined as the absolute number and percentage of different definitions for a given RRC nomenclature, which will be reported in a table. Overlap was defined as the percentage of definitions that were used to describe two or more different RRC nomenclatures.

A sub-analysis identified RRC steps in Western (including Australia, Russia and Turkey) vs Asian countries and in different time periods (2009–2015 vs 2016–2021) in an effort to detect geographical or temporal peculiarities/tendencies.

### Data extraction and quality assessment (Fig. S1)

Each article was carefully read and analysed independently by two authors (B.S. and L.S.) in an effort to identify surgical steps that authors attributed specifically to RRC as opposed to a minimal/standard right colectomy.

Study quality was assessed using Newcastle Ottawa Scale (NOS) for non-RCTs and the modified Jadad scale score for RCTs.

NOS is an assessment tool used to measure the quality of non-randomized studies included in systematic reviews [14]. Each article was assessed for 9 parameters, each awarding up to one point, with a maximum total score of 9 points. Modified Jadad scale score is used to assess the quality of RCT by evaluating three parameters each awarding one point with three point awarded for high-quality RCT [15].

### Data synthesis (Fig. S1)

The techniques described in each article were listed based on the presence or absence of each of the steps previously identified. These data were grouped in an excel sheet.

Furthermore, the definition of each technique given by the original author was recorded to reveal overlapping of definitions and evaluate heterogeneity.

Descriptive statistics were produced from the dataset: categorical data were merged and are reported as numbers and/or percentages. There was no comparative statistical analysis.

## Results

### Systematic search

**Fig. 1:**
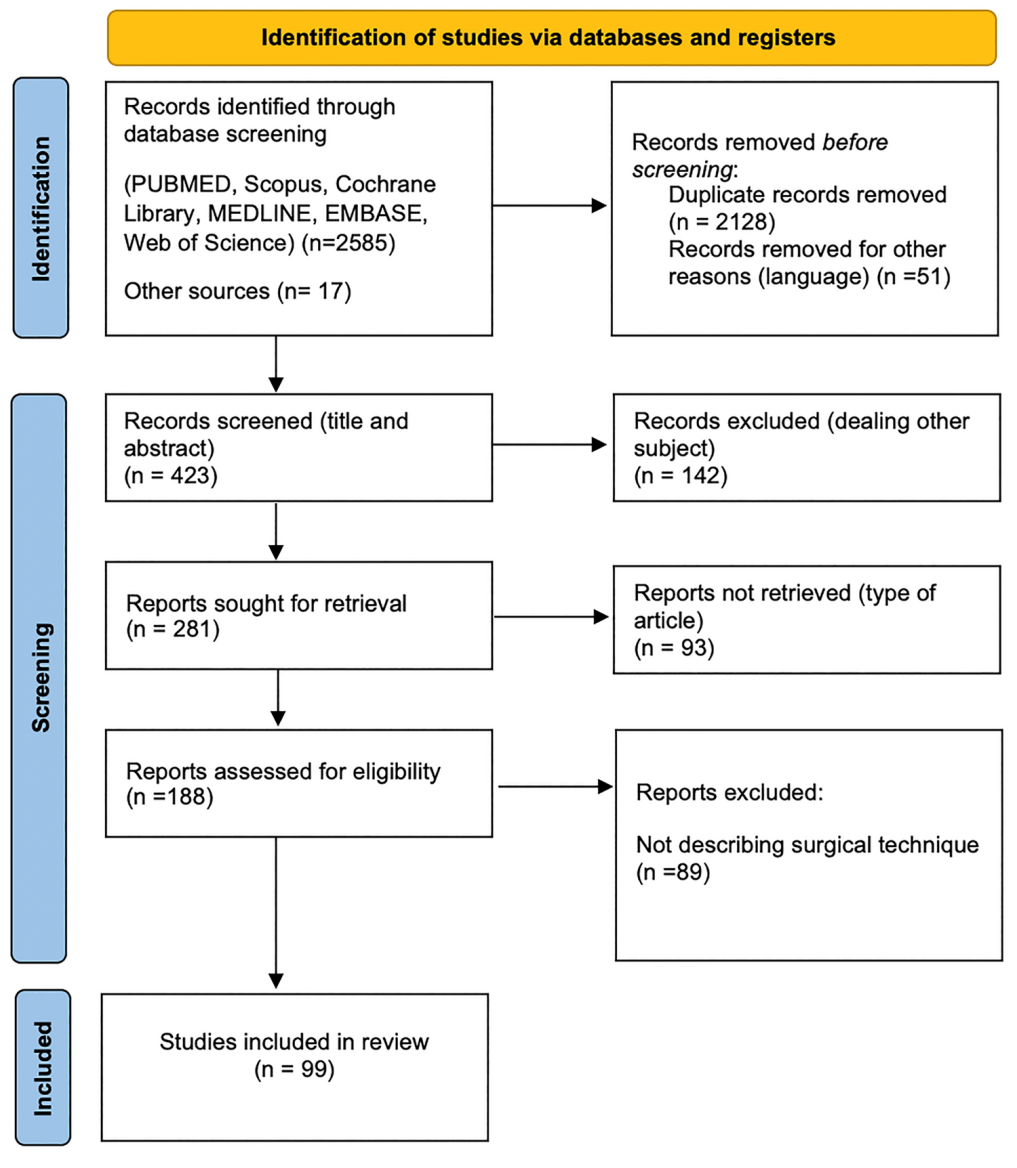
PRISMA 2020 flow-chart for the systematic search process

The systematic search process is summarised in Fig. 1. The initial database search identified 2602 articles. After initial screening and exclusion of duplications and after full-text reading of the remaining articles, 99 eligible articles were included in the qualitative review.

### Quality assessment

Table 1 summarizes year, journal, design and country of publication for each study as well as NOS or Jadad scale score [16–113]. All studies were published between 2009 and 2021 and > 50% from 2018 onwards. The most common study type was retrospective (54%), while only 3% of the studies included were RCTs. Average NOS score was 7.8 and average modified Jadad scale score was 6.7.

**Table 1:** Included studies and NOS/Jadad assessment

First author	Year	Journal	Study	Country	NOS/Jadad Score
Alharbi RA et al. [16]	2020	Annals of Saudi Medicine	Retrospective study	Saudi Arabia	7
Alhassan N et al. [17]	2019	Surgical Endoscopy	Systematic review and pooled analysis	Canada	8
Bae SU et al. [18]	2018	International Journal of Colorectal Disease	Retrospective study	South Korea	9
Balciscueta z et al. [19]	2021	European Journal of Surgical Oncology	Meta-analysis	Spain	7
Benz S et al. [20]	2018	Techniques in Coloproctology	Prospective study	Germany	9
Bernhoff R et al. [21]	2017	Colorectal Disease	Retrospective study	Sweden	8
Bertelsen CA et al. [22]	2018	Diseases of the Colon & Rectum	Retrospective study	Denmark	8
Ceccarelli G et al. [23]	2020	Surgical Endoscopy	Retrospective study	Italy	7
Chaouch MA et al. [24]	2019	World Journal of Surgery	Systematic review and meta-analysis	Tunisia	8
Dai W et al. [25]	2018	Cancer Management and Research	Retrospective study	China	7
Daniels M et al. [26]	2015	International Journal of Colorectal Disease	Prospective study	Germany	8
Du S et al. [27]	2018	Surgical Endoscopy	Retrospective study	China	7
Ehrlich A et al. [28]	2016	Scandinavian Journal of Surgery	Retrospective study	Finland	7
Elias AW et al. [29]	2018	Journal of Gastroenterology Surgery	Retrospective study	USA	7
Esch JS et al. [30]	2019	BMC Surgery	Retrospective study	Germany	7
Feng B et al. [31]	2012	Surgical Endoscopy	Retrospective study	China	8
Feng B et al. [32]	2014	Surgical Endoscopy	Randomised controlled trial	China	8*
Furnes B et al. [33]	2018	Scandinavian Journal of Surgery	Retrospective study	Norway	7
Galizia G et al. [34]	2013	International Journal of Colorectal Disease	Prospective study	Italy	9
Gao Z et al. [35]	2018	Annals of surgery	Randomised controlled trial	China	7*
Gaupset R et al. [36]	2018	Journal of laparoendoscopic & advanced surgical techniques	Retrospective study	Norway	7
Gouvas N et al. [37]	2012	Colorectal Disease	Prospective study	Greece	8
Hamzaoglu I et al. [38]	2018	Techniques in coloproctology	Retrospective study	Turkey	9
Han DP et al. [39]	2013	International Journal of Colorectal Disease	Retrospective study	China	8
Han DP et al. [40]	2014	Surgery today	Retrospective study	China	7
He Z et al. [41]	2019	Surgical Endoscopy	Retrospective study	China	7
Ho MLL et al. [42]	2019	Journal of Gastrointestinal Oncology	Technical note	China	8
Hohenberger W et al. [9]	2009	Colorectal Disease	Prospective study	Germany	9
Huang JL et al. [43]	2015	International Journal of surgery	Retrospective study	China	8
Kanemitsu Y et al. [44]	2013	Diseases of the Colon & Rectum	Retrospective study	Japan	9
Karachun A et al. [45]	2019	British Journal of Surgery	Randomised controlled trial	Russia	6*
Kataoka K et al. [46]	2020	British Journal of Surgery	Retrospective study	Japan	8
Killeen s et al. [47]	2014	Colorectal Disease	Systematic Review	Ireland	8
Killeen S et al. [48]	2014	Techniques in Coloproctology	Technical note	USA	
Kim CW et al. [49]	2016	Medicine	Observational study	South Korea	7
Kim IY et al. [50]	2016	International Journal of Surgery	Retrospective study	South Korea	9
Kim NK et al. [51]	2016	Surgical Oncology	Technical note	South Korea	8
Kim JS et al. [52]	2021	Asian Journal of Surgery	Retrospective study	South Korea	7
Kobayashi H et al. [53]	2020	Clinics in Colon and Rectal Surgery	Retrospective study	Japan	9
Koc MA et al. [54]	2021	Medicine	Retrospective study	Turkey	7
Lan YT et al. [55]	2010	Annals of Surgical Oncology	Retrospective study	Taiwan	9
Larach JT et al. [56]	2021	ANZ Journal of Surgery	Retrospective study	Australia	7
Lee SD et al. [57]	2009	International Journal of Colorectal Disease	Retrospective study	South Korea	9
Lee JM et al. [58]	2020	Diseases of the Colon & Rectum	Retrospective study	South Korea	9
Li J et al. [59]	2020	The International Journal of Medical Robotics and Computer Assisted Surgery	Retrospective study	China	8
Liang JT et al. [60]	2015	Surgical Endoscopy	Prospective study	Taiwan	7
Livadaru C et al. [61]	2019	World Journal of Gastrointestinal Oncology	Retrospective study	Romania	7
Luglio G et al. [62]	2015	Annals of Medicine and Surgery	Prospective study	Italy	8
Melich G et al. [63]	2014	Canadian Journal of Surgery	Retrospective study	South Korea	7
Merkel S et al. [64]	2016	British Journal of Surgery	Observational study	Germany	9
Mori S et al. [65]	2015	Digestive Surgery	Observational study	Japan	7
Mori S et al. [66]	2014	Surgical Endoscopy	Retrospective study	Japan	7
Nagasaki T et al. [67]	2015	World Journal of Surgery	Observational study	Japan	8
Nakajima K et al. [68]	2014	Japanese Journal of Clinical Oncology	Randomised controlled trial	Japan	7*
Olmi S et al. [69]	2020	Journal of the Society of Laparoscopic & Robotic Surgeons	Retrospective study	Italy	7
Olofsson F et al. [70]	2016	Colorectal Disease	Retrospective study	Sweden	8
Ouyang M et al. [71]	2019	Cancer Management and Research	Retrospective study	China	7
Ow ZGW et al. [72]	2020	European Journal of Surgical Oncology	Systematic Review and Meta-Analysis	Singapore	9
Ozben V et al. [73]	2018	Journal of Robotic Surgery	Prospective study	Turkey	7
Pedrazzani C et al. [74]	2018	Journal of Gastrointestinal Surgery	Retrospective Study	Italy	
Perrakis A et al. [75]	2018	Archives of Medical Science	Retrospective study	Greece	7
Petz W et al. [76]	2017	European Journal of Surgical Oncology	Prospective study	Italy	8
Pramateftakis MG et al. [77]	2010	Techniques in coloproctology	Clinical study	Greece	7
Prevost GA et al. [78]	2018	World Journal of Surgical Oncology	Retrospective study	Switzerland	8
Ramachandra C et al. [79]	2020	Indian Journal of Surgical Oncology	Retrospective study	India	7
Rinne JKA et al. [80]	2019	Journal of Gastrointestinal Surgery	Retrospective study	Finland	9
Sahara K et al. [81]	2020	Surgery Today	Retrospective study	Japan	7
Sammour T et al. [82]	2019	Colorectal Disease	Retrospective study	USA	8
Sheng QS et al. [83]	2017	Annals of Surgical Treatment and Research	Retrospective study	China	7
Shin JW et al. [84]	2014	Techniques in Coloproctology	Retrospective study	South Korea	9
Shin JK et al. [85]	2018	Surgical Endoscopy	Retrospective study	South Korea	8
Siani LM et al. [86]	2014	Scandinavian Journal of Surgery	Retrospective study	Italy	7
Siddiqi N et al. [87]	2020	Surgical Endoscopy	Retrospective study	UK	8
Spinoglio G et al. [88]	2016	Annals of Surgical Oncology	Retrospective study	Italy	8
Spinoglio G et al. [89]	2018	Annals of Surgical Oncology	Retrospective study	Italy	8
Storli KE et al. [90]	2013	Digestive Surgery	Prospective study	Norway	7
Storli KE et al. [91]	2014	Techniques in Coloproctology	Prospective study	Norway	9
Subbiah R et al. [92]	2015	International Journal of Colorectal Disease	Retrospective study	India	9
Takahashi H et al. [93]	2016	Surgery today	Retrospective study	Japan	7
Takemasa I et al. [94]	2013	Surgical Endoscopy	Prospective study	Japan	7
Thorsen Y et al. [95]	2016	Techniques in Coloproctology	Prospective study	Norway	8
Thorsen Y et al. [96]	2019	ScienceDirect	Observational study	Norway	7
Tominaga T et al. [97]	2021	International Journal of Clinical Oncology	Observational study	Japan	8
Wang Y et al. [98]	2017	World Journal of Surgical Oncology	Observational study	China	8
Wei M et al. [99]	2018	Medicine	Observational study	China	7
West NP et al. [100]	2010	Diseases of the Colon & Rectum	Original article	Denmark	9
Willard CD et al. [101]	2018	International Journal of Colorectal Disease	Observational study	Norway	9
Wu QB et al. [102]	2016	Surgical Endoscopy	Prospective study	China	8
Wu H et al. [103]	2020	Journal of the Balkan Union of Oncology	Retrospective study	China	7
Xie D et al. [104]	2016	Annals of Surgical Oncology	Observational study	China	8
Yamamoto M et al. [105]	2019	Surgical Endoscopy	Prospective study	Japan	7
Yan D et al. [106]	2020	Journal of the Balkan Union of Oncology	Retrospective study	China	7
Yang Y et al. [107]	2019	Diseases of the Colon & Rectum	Technical notes	USA	7
Yi X et al. [108]	2019	Surgical Endoscopy	Retrospective study	China	7
Yozgatli TK et al. [109]	2019	Journal of Laparoendoscopic & Advanced Surgical Techniques	Observational study	Turkey	7
Zedan A et al. [110]	2021	International Surgery of Surgical Oncology	Prospective study	Egypt	8
Zhao LY et al. [111]	2014	World Journal of Gastroenterology	Prospective study	China	9
Zhao LY et al. [112]	2014	World Journal of Gastroenterology	Retrospective study	China	8
Zurleni T et al. [113]	2018	International Journal of Colorectal Disease	Retrospective study	Italy	9

*Jadad score

### Primary aim: RRC surgical steps

Eight surgical steps were identified and recorded as specific for RRC as opposed to standard right colectomy:

1. Central arterial ligation (at the root from the superior mesenteric artery (SMA)).
2. Preservation of mesocolic integrity.
3. Dissection along the superior mesenteric vein (SMV) plane.
4. Dissection along the left border of the SMA.
5. Dissection of the gastrocolic trunk of Henle (GCTH).
6. Sub-pyloric lymph-nodes dissection.
7. Complete Kocher’s manoeuvre.
8. Omentectomy.

Central arterial ligation was described in 100% of the included studies; preservation of mesocolic integrity in 73%; dissection along the SMV plane in 67%; dissection along the left border of the SMA in 11%; dissection of the GCTH in 45%; sub-pyloric lymph-nodes dissection in 18%; a complete Kocher’s manoeuvre in 11% and an omentectomy in 39% of studies.

### Primary aim: RRC nomenclature

Analysis of nomenclature identified six RRC techniques: complete mesocolic excision (CME), complete mesocolic excision with central vascular ligation (CME + CVL), central vascular ligation (CVL), modified complete mesocolic excision (mCME), D3 lymphadenectomy (D3) and complete mesocolic excision with D3 lymphadenectomy (CME + D3).

### Primary aim: number of reports and prevalence of each surgical step for a given technique

1. CME (n of studies = 48)

All CMEs studies reported central arterial ligation but not all the papers clearly reported preservation of mesocolic integrity (83.3%) and SMV dissection (66.7%). GCTH dissection was associated in 35.4%, sub-pyloric lymph-nodes dissection in 20.8%, omentectomy in 41.7% and a full Kocher’s manoeuvre in 12.5%.

(2) CME + CVL (n = 22)

CME + CVL descriptions included preservation of mesocolic integrity in 83.3%, SMV dissection in 54.5% and SMA dissection in 9.1%. GCTH dissection was described in 40.9%, sub-pyloric nodes retrieval in 18.2%, omentectomy in 45.5% and a full Kocher’s manoeuvre in 13.6%.

(3) CVL (n = 1)

CVL only: this paper described central arterial ligation only.

(4) Modified CME (mCME, n = 5)

mCME is a “modified technique” of CME that included preservation of mesocolic integrity, reported in 80% and SMV dissection in 60%. GCTH dissection was reported in 60% of the papers, sub-pyloric nodes retrieval in 20% and omentectomy in 40%. Dissection along the SMA or a full Kocher’s manoeuvre was not reported.

(5) D3 (n = 18)

D3 studies included preservation of mesocolic integrity in 33.30%, dissection of the SMV in 83.3% and of the SMA in 38% of reports. Dissection of GCTH and sub-pyloric nodes were reported in 66.6% and 16.7%, respectively; omentectomy and a full Kocher’s manoeuvre in 22.2% and in 5.5%, respectively.

(6) CME + D3 (n = 5)

CME + D3 studies included mesocolic preservation in 100%, dissection along the SMV in 80%, along the SMA in 0%, of the GCTH in 80%, of the sub-pyloric nodes in 0%, a full Kocher manoeuvre in 0% and omentectomy in 40%.

Results of systematic analysis of surgical techniques are summarised in Table 2.

**Table 2:** Percentage (%) of surgical steps reported for each procedure

	Central arterial ligation %	Preservation Of mesocolic integrity %	Dissection along SMV %	Dissection along SMA %	Dissection Of GCTH %	Sub-pyloric dissection	Complete kocher manoeuvre %	Omentectomy %
ALL (99)	100.0	73.0	67.0	11.0	45.0	18.0	11.0	39.0
CME (48)	100.0	83.3	66.7	4.2	35.4	20.8	12.5	41.7
CME + CVL (22)	100.0	81.8	54.5	9.1	40.9	18.2	13.6	45.5
CVL (1)	100.0	0.0	0.0	0.0	0.0	0.0	0.0	0.0
MCME (5)	100.0	80.0	60.0	0.0	60.0	20.0	0.0	40.0
D3 (18)	100.0	33.3	83.3	38.9	66.6	16.7	5.5	22.2
CME + D3 (5)	100.0	100.0	80.0	0.0	80.0	0.0	0.0	40.0

*CME* complete mesocolic excision; *CVL* central vascular ligation; *MCME* modified complete mesocolic excision; *SMV* superior mesenteric vein; *SMA* superior mesenteric artery; *GCTH* gastrocolic trunk of Henle

### Secondary aim: heterogeneity in definitions

Thirty-five different definitions of RRC were identified (Table 3). The definitions used in each study are reported in Table S2 [16–113]. Among the forty-eight articles regarding CME, there were twenty-two different descriptions of the operation. The most common definitions (recurring in 16.67% of studies) were central arterial ligation and preservation of mesocolic integrity. CME + CVL featured fourteen different definitions in twenty-two studies, the most common of which (35.71%) included only central arterial ligation and conservation of mesocolic integrity. The modified version of CME (mCME) was defined in four different ways. D3 was described with eleven different techniques: the most common technique (22.22%) included CVL, mesocolic preservation, SMV dissection, gastrocolic and pyloric nodes dissections and omentectomy. D3 + CME featured five descriptions, in 40% of cases including CVL, mesocolic preservation, SMV, gastrocolic and pyloric nodes dissection.

**Table 3:** RRC definition based on each nomenclature

Surgical step	CME (48)	CME + CVL (22)	CVL (1)	Modified CME (5)	D3 + CME (5)	D3 (18)	ALL (99) (%)
1	4.2%	9.1%	100%	–	–	5.5%	6
1, 2	6.6%	22.7%	–	20%	–	–	15
1, 3	4.2%	–	–	20%	–	16.6%	6
1, 6	–	–	–	–	–	5.5%	1
1, 7	–	4.5%	–	–	–	–	1
1, 8	2%	–	–	–	–	–	1
1, 2, 3	10.4%	–	–	–	–	5.5%	6
1, 2, 5	4.2%	–	–	–	20%	–	3
1, 4, 5	–	–	–	–	–	5.5%	1
1, 5, 7	2%	–	–	–	–	–	1
1, 2, 8	2%	4.5%	–	–	–	–	2
1, 5, 8	2%	–	–	–	–	–	1
1, 2, 3, 4	2%	–	–	–	–	–	1
1, 2, 3, 5	8.2%	13.6%	–	–	40%	5.5%	10
1, 2, 3, 7	4.2%	–	–	–	–	–	2
1, 2, 3, 8	–	9.1%	–	–	20%	–	3
1, 2, 5, 6	–	–	–	20%	–	–	1
1, 2, 5, 8	–	4.5%	–	–	–	–	1
1, 2, 6, 8	2%	–	–	–	–	–	1
1, 3, 4, 5	–	–	–	–	–	22.2%	4
1, 3, 5, 6	–	–	–	–	–	5.5%	1
1, 3, 5, 8	–	4.5%	–	–	–	–	1
1, 2, 3, 5, 6	2%	–	–	–	–	–	1
1, 2, 3, 5, 8	4.2%	4.5%	–	40%	20%	16.6%	9
1, 2, 3, 6, 8	2%	–	–	–	–	–	1
1, 2, 3, 7, 8	8.2%	4.5%	–	–	–	–	5
1, 3, 4, 5, 8	–	–	–	–	–	5.5%	1
1, 3, 5, 6, 8	2%	4.5%	–	–	–	–	2
1, 2, 3, 4, 5, 6	–	4.5%	–	–	–	–	1
1, 2, 3, 5, 6, 8	6.2%	–	–	–	–	–	3
1, 2, 3, 5, 7, 8	4.2%	–	–	–	–	–	2
1, 2, 3, 6, 7, 8	4.2%	4.5%	–	–	–	–	2
1, 2, 3, 4, 5, 6, 7	–	–	–	–	–	5.5%	1
1, 2, 3, 4, 5, 6, 8	2%	4.5%	–	–	–	–	2

1 central vascular ligation 2 conservation of mesocolic integrity 3 dissection along SMV 4 dissection along SMA 5 dissection of GCTH 6 sub-piloric node dissection 7 full Kocker manoeuvre 8 omentectomy

*CME* complete mesocolic excision; *CVL* central vascular ligation

### Secondary aim: overlap in definitions

Forty percent of the definitions were used to identify more than one RRC technique. Regarding CME, 36.36% of definitions were unique, while the rest overlapped with definitions used for CME + CVL (40.90%), D3 (22.72%), mCME (13.64%), and D3 + CME (13.64%). For what concerns CME + CVL, 28.57% of definitions were unique, while the rest overlapped with CME (64.29%), D3 (14.29%), mCME (14.29%) or D3 + CME (21.42%). For mCME, 75% of definitions overlapped with CME, 50% with CME + CVL, 50% with D3 and 25% with D3 + CME. D3 + CME had no unique definition, with 75% overlap with CME, 75% with CME + CVL, 25% with mCME and 50% with D3. D3 had 54.54% of unique definitions, while others overlapped with CME (45.45), CME + CVL (27.27%), mCME (18.18%), D2 + CME (18.18%).

### East vs West

All six RRC steps were used by both Easter and Western studies. Of note, omentectomy was more prevalent in Eastern studies (48% vs 30.6%) as was GCTH dissection (54% vs 36,7%), while sub-pyloric lymph-node dissection was more common in the West (14% vs 22,4%), and dissection along the left border of the SMA was almost three times more common in the west (6 vs 16,3%) (Fig. S2).

### RRC over time

All six RRC steps were used in both periods. Of note, omentectomy (32,3% vs 42,6%), dissection along the SMV (58,1% vs 70,6%) and dissection of GCTH (35,5% vs 50,0%) were more prevalent in more recent time (Fig. S3).

## Discussion

The current systematic review identified significant variability in the reporting and definitions of RRC, despite the existence of standardised, systematic descriptions that have been produced over years. Up to 35 different combinations of the key components of a RRC were observed, with several studies inappropriately claiming to perform a given procedure according to the descriptions provided by the authors. Such variability raises several concerns, as it is difficult to address the actual benefits of extensive approaches when no agreed terminology and procedures are being adopted.

Since the detailed description of D3 lymphadenectomy advocated by Asian guidelines [114] and the report on CME with CVL by Hohenberger et al. [9] to perform a RRC, a vast myriad of articles with a combination of definitions of RRC have been published.

The lack of uniformity undermines the proper evaluation of the clear benefits of any technique over the others. It is interesting to note that the CME description by Hohenberger [9] clearly differs from any “standard” right hemicolectomy for right colon cancer, but some of the proposed techniques do not differ from a proper right colectomy for cancer. Even if some authors have suggested some benefits of extended lymphadenectomy [115], most agree that there is need for more prospective or randomised studies to identify this as  necessary for RRC [116]. The discrepancies in available definitions used in the published studies make it difficult to draw conclusions.

This systematic review offers several contributions to the understanding of RRC. It identifies the fundamental surgical steps reported by every single study. Commonly used definitions of these steps can be found in Table 4. Some of these surgical steps are adopted quite uniformly by all the authors, while others seem not to be considered fundamental.

**Table 4:** Common definitions of the surgical steps identified for radical right colectomy

Step	Definition
(1): Central arterial ligation	Ligation at their roots of the ileocolic artery, the right colic artery (when present) and the right branch of the middle colic artery (for cancers of the caecum and ascending colon up to the right flexure) or the stem of the middle colic artery (cancers of the left side of the hepatic flexure or proximal transverse colon)
(2): Preservation of mesocolic integrity	Dissection along the embryological plane and complete excision of the mesocolon, conserving the integrity of its anterior and posterior sheaths
(3) Dissection along the superior mesenteric vein (SMV) plane	The dissection plane is offered by the anterior and lateral face of the SMV
(4) Dissection along the left border of the superior mesenteric artery (SMA)	The dissection plane offered by the SMA run below and laterally to the SMV. It requires a dissection of the left border of the SMV and the anterior surface of the aorta
(5) Dissection of the gastrocolic trunk of Henle (GCTH)	The GCTH has numerous and frequent anatomic variations. In most cases the right/middle colic vein can be dissected free and individually divided while preserving pancreaticoduodenal and gastroepiploic veins. Further lymph nodes are harvested at this level
(6) Sub-pyloric lymph-nodes dissection	Removal of lymphoadipose tissue around the origin of the gastroepiploic vessels. This manoeuvre usually includes sacrifice of these vascular structures
(7) Complete Kocher’s manoeuvre	Complete mobilisation of the 1^st^ to 3^rd^ portions of the duodenum from their attachments to achieve 180° rotation of the duodenum and pancreatic head to access retropancreatic and caval lymph nodes
(8) Omentectomy	Resection of the greater omentum together with the surgical specimen

The main surgical steps commonly shared by the authors are two, central arterial ligation and preservation of the mesocolic integrity. Central arterial ligation ensures harvesting of all nodes along the colon’s feeding vessels (ileocolic vessels and right branch of the middle colic vessels in RRC).

It allows a significantly higher number of nodes to be excised compared to so-called low-tie of the organ’s vessels. This technique indeed may provide rationale for superior oncological results (in terms of both local and distal control) [9] but certainly it is not a novel concept; high-tie of vascular structures being one of the pillars of oncologic surgery. The rationale to remove more lymph nodes is also suggested by reports on lymph node ratio (number of positive nodes divided by the total number of harvested nodes) that can be more prognostically relevant than the number of positive nodes per se [117]. Preservation of mesocolic integrity is predominantly mentioned in studies focussing on CME and it can be properly regarded as a “novel” manoeuvre; it follows a well-known anatomical dissection plane and encompasses the removal of all the lymphoadipose tissue lateral to the SMV. The embryologic *fasciae* that need to be respected during RRC with CME would be the fusion *fascia* of Toldt and the fusion *fascia* of Fredet [118–120].

Whether the integrity of the mesocolic fascia does represent a necessity to prevent local recurrence is far from being clarified. The proposers of CME should be credited for having raised attention towards the importance of a truly radical approach to right colon cancers [9]

A retrospective study of surgical specimens reported longer survival for those patients with stage III colon cancer whose colon was excised with intact mesocolon, compared with patients who had received less than optimal surgery. The surgical technique is well defined and requires the surgeon (1) to remain within the mesocolic plane, (2) to perform central ligation of the tumour-feeding artery, and (3) to remove an appropriate length of large bowel on either side of the tumour [100]. A medial to lateral approach to dissection has been advocated with laparoscopy and a lateral to medial one in open surgery, but the direction of dissection was independent from extent of resection and never reported as specific to RRC.

According to Hohenberger et al. [9], the lymphoadipose tissue covering the SMV and the head of the pancreas should be removed in the event of potentially affected nodes at preoperative CT scan, or if these are detected intraoperatively at these sites. The removal of the lymphoadipose tissue along both lateral and medial sides of SMV and the GCTH defines a D3 lymphadenectomy [8, 121, 122].

For what concerns the other surgical steps variably associated to RRC, the consensus drops significantly, and they are reported by a minority of authors.

The dissection along the SMV between the ileocolic vein and the GCTH (Gillot’s fat pad) [123] is based on data suggesting that 3% of right colon cancer metastasise to central lymph nodes, located anteriorly to the SMV [19, 117, 128–131]. This may be important in the staging process (as up to 0.2–2% of patients harbour skip metastases in central nodes) and might probably ameliorate prognosis [117, 128, 129]. The SMV plane of dissection is an excellent surgical plane for dissection. Nevertheless, it can be considered dangerous due to the importance of the structure and because of the thin wall of the vein [132–134].

Authors reporting on the more extensive D3 lymphadenectomy most frequently mention dissection of the SMA. This procedure may result in autonomic dysfunction, due to consensual resection of nerve plexuses lying anterior to the SMA. Symptoms may include severe refractory diarrhoea [94, 95].

Dissection of the GCTH requires the removal of lymphoadipose tissue covering the head of the pancreas and is usually employed by authors of D3 or in case of tumours of the hepatic flexure or proximal transverse colon. No study to date has specifically focussed on the advantages of this surgical step alone.

Dissection of sub-pyloric lymph nodes, complete Kocher manoeuvre and omentectomy are generally not considered integral part of RRC if not in a limited number of reports. Dissection of station six nodes could be theoretically useful in cancers of the hepatic flexure and proximal transverse colon [135]. As said, no benefit has been demonstrated and there is no consensus to its routine application. A complete Kocher manoeuvre allows dissection of retro-duodenopancreatic nodes, but no rationale exists for their removal in colon cancer. The utility of omentectomy in colonic surgery has not been thoroughly investigated to date.

Different authors with variable combinations of the main surgical steps, resulting in a great heterogeneity of definitions, have defined individual surgical techniques. In this systematic review, 36.36% of CME definitions were unique, while the rest overlapped with definitions used for CME + CVL (40.90%), D3 (22.72%), mCME (13.64%) and D3 + CME (13.64%).

Obviously, this variability in definition makes aggregation of results from these studies incorrect from a methodological point of view, such that meta-analyses would be of questionable scientific value. In fact, the current “CME” literature includes different surgical operations, which are mistakenly given the same name.

Of course, this introduces a further element of confusion in interpretation of the literature, making comparison among different RRC techniques virtually impossible and the twelve ongoing randomized trials possibly not completely confrontable. Of note, there have been proposals for standardised assessment and reporting of CME and D3 lymphadenectomy in RRC; a consistent utilisation of such approaches could ease the interpretation of prospective studies, allowing to objectively addressing whether extended approaches confer any survival benefit [136, 137].

After more than 10 years of debate, it is apparent that a clarification on surgical technique has been long overdue: a globally agreed consensus on the precise surgical steps to be performed for each given procedure (herein defined RRC) is necessary and expectedly awaited.

## Conclusions

Central arterial ligation is unanimously considered indispensable to perform RRC for right colon cancer. Other surgical steps are more debated; preservation of mesocolic integrity has clearly a central role in CME and dissection along the SMV in D3. There is great heterogeneity and consistent overlap among definitions of all RRC techniques. Confusion in the definition of a RRC might jeopardise the reliability of available results, limiting the generalizability, and making comparisons difficult. Consensus definitions are warranted to usher progress in right colon cancer surgery.

## Supplementary Information

Below is the link to the electronic supplementary material.

Figure S1: Data extraction and synthesis (TIF 314 kb)

Figure S2: RRC-steps in Eastern vs Western studies (TIF 84 kb)

Figure S3: RRC-steps in older (2009-2015) and more recent (2016-2021) time periods (TIF 88 kb)

Supplementary file4 (DOCX 17 kb)

Supplementary file5 (DOCX 16 kb)
