# Correction: Definition and reporting of lymphadenectomy and complete mesocolic excision for radical right colectomy: a systematic review

**DOI:** 10.1007/s00464-022-09747-0

**Published:** 2022-11-04

**Authors:** Giuseppe S. Sica, Danilo Vinci, Leandro Siragusa, Bruno Sensi, Andrea M. Guida, Vittoria Bellato, Álvaro García-Granero, Gianluca Pellino

**Affiliations:** 1 Minimally Invasive Unit, Department of Surgical Science, University Tor Vergata, Rome, Italy; 2 Department of Surgical Science, Policlinico Tor Vergata – University Tor Vergata, Rome, Italy; 3 Ospedale IRCCS San Raffaele, Milan, Italy; 4 Colorectal Unit, Hospital Universitario Son Espases, Palma, Spain; 5 Applied Surgical Anatomy Unit, Human Embryology and Anatomy Department, University of Valencia, Valencia, Spain; 6 Human Embryology and Anatomy Department, University of Islas Baleares, Palma, Spain; 7 Department of Advanced Medical and Surgical Sciences, Università Degli Studi Della Campania “Luigi Vanvitelli”, Naples, Italy; 8 Colorectal Surgery, Vall d’Hebron University Hospital, Barcelona, Spain

## Correction to: Surgical Endoscopy 10.1007/s00464-022-09548-5

This article was updated to correct the CME+ D3 data in the text and in Table 2.

