# Supplementary figures and images for: Definition and reporting of lymphadenectomy and complete mesocolic excision for radical right colectomy: a systematic review
Source: Surg Endosc. 2022 Sep 12;37(2):846–61. doi: 10.1007/s00464-022-09548-5 (PMC9944740; doi:10.1007/s00464-022-09548-5)

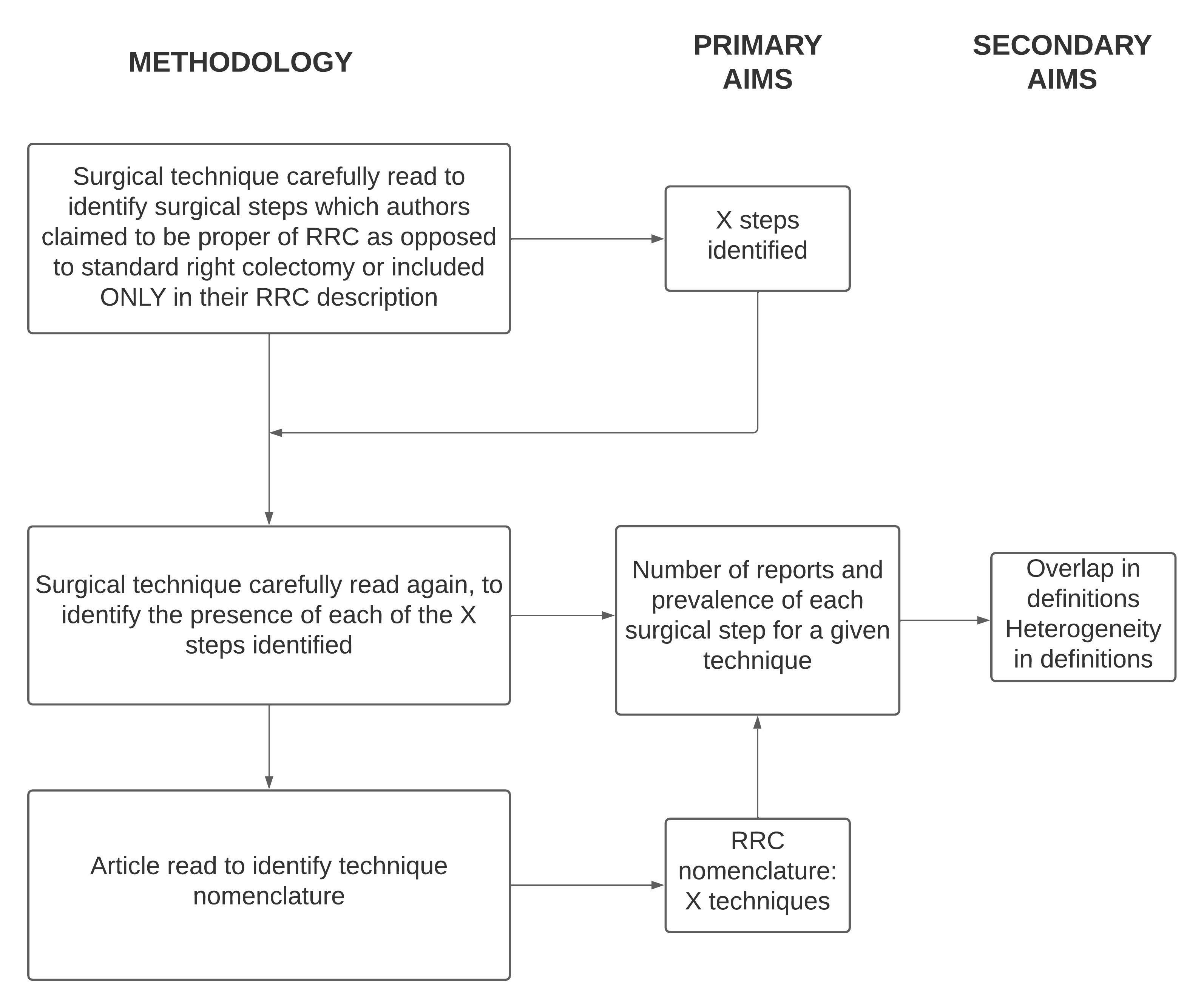

Supplement: Supplementary file 1 — Figure S1: Data extraction and synthesis (TIF 314 kb) [file 464_2022_9548_MOESM1_ESM.tif]

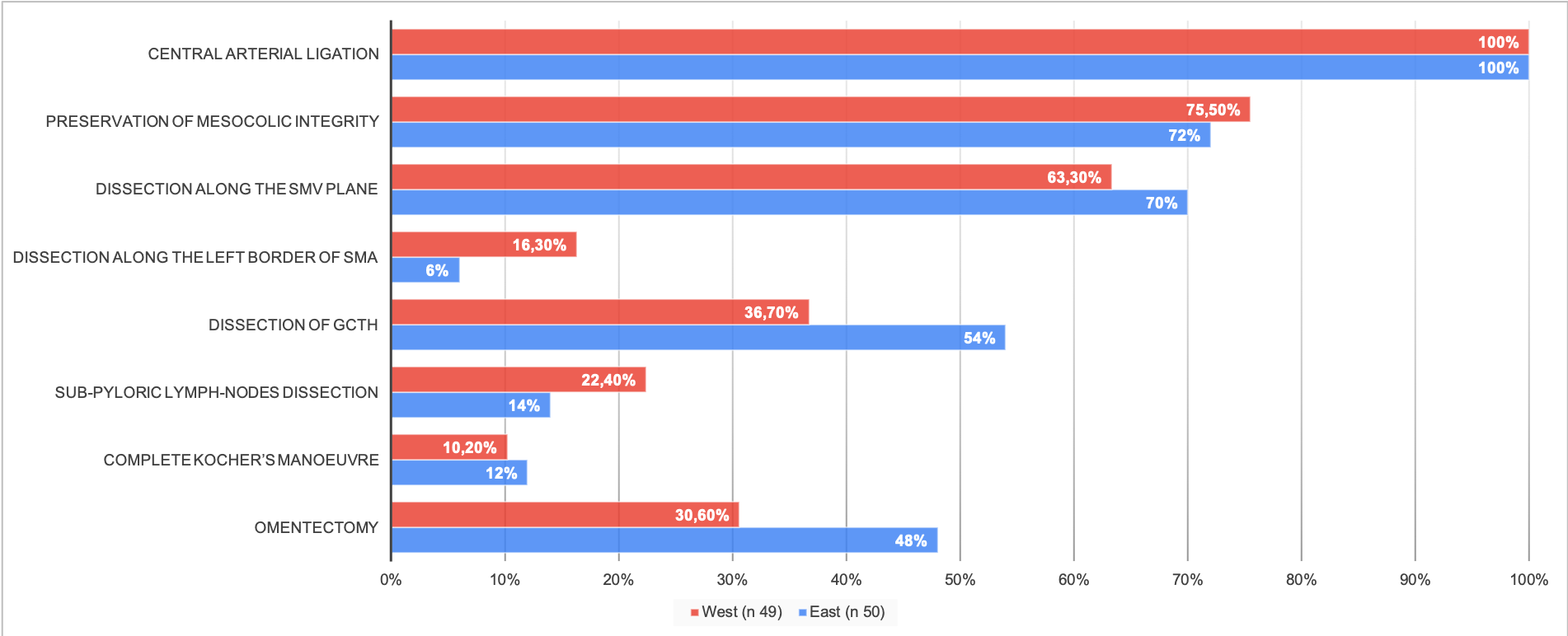

Supplement: Supplementary file 2 — Figure S2: RRC-steps in Eastern vs Western studies (TIF 84 kb) [file 464_2022_9548_MOESM2_ESM.tif]

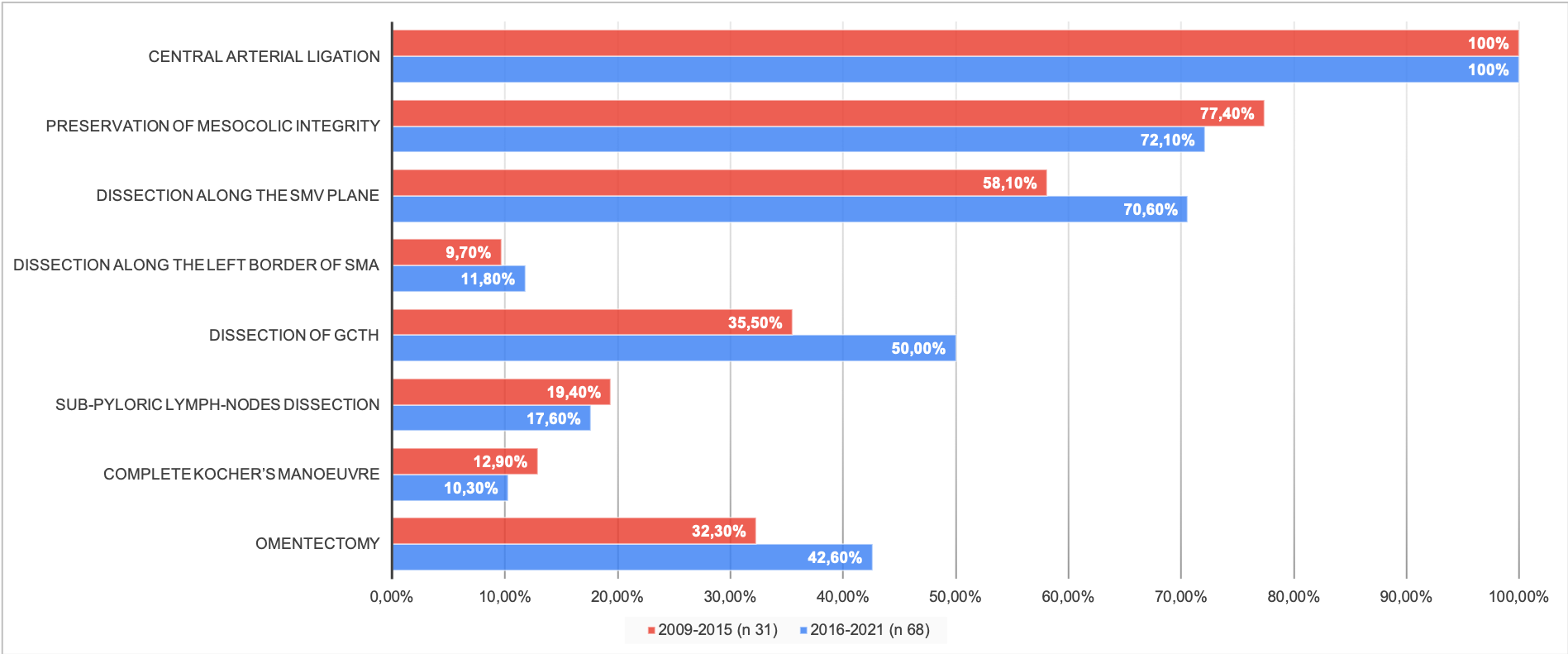

Supplement: Supplementary file 3 — Figure S3: RRC-steps in older (2009-2015) and more recent (2016-2021) time periods (TIF 88 kb) [file 464_2022_9548_MOESM3_ESM.tif]
