# Supplementary material for: Definition and reporting of lymphadenectomy and complete mesocolic excision for radical right colectomy: a systematic review
Source: Surg Endosc. 2022 Sep 12;37(2):846–61. doi: 10.1007/s00464-022-09548-5 (PMC9944740; doi:10.1007/s00464-022-09548-5)
Supplement: Supplementary file 5 — Supplementary file5 (DOCX 16 kb) [file 464_2022_9548_MOESM5_ESM.docx]

**Table S2:** Definitions of RRC in each study

| First author | Year | Nomenclature used | Technique reported |
| --- | --- | --- | --- |
| Alharbi RA et al [16] | 2020 | CME | 1,2,3,6,7,8 |
| Alhassan N et al [17] | 2019 | CME | 1,2 |
| Bae SU et al [18] | 2018 | CME+CVL | 1,2,3,5,6,8 |
| Balciscueta z et al [19] | 2021 | D3+CME | 1,2,3,5 |
| Benz S et al [20] | 2018 | CME | 1,2,3 |
| Bernhoff R et al [21] | 2017 | CME | 1,2,3 |
| Bertelsen CA et al [22] | 2018 | CME | 1 |
| Ceccarelli G et al [23] | 2020 | CME | 1,2,3,5,8 |
| Chaouch MA et al [24] | 2019 | CME | 1,2,3 |
| Dai W et al [25] | 2018 | D3 | 1,4,5 |
| Daniels M et al [26] | 2015 | CME | 1,2,3,7,8 |
| Du S et al [27] | 2018 | CME | 1,2,3,5,6,8 |
| Ehrlich A et al [28] | 2016 | CME+CVL | 1 |
| Elias AW et al [29] | 2018 | CVL | 1 |
| Esch JS et al [30] | 2019 | CME | 1,2,3,5 |
| Feng B et al [31] | 2012 | CME | 1,2,6,8 |
| Feng B et al [32] | 2014 | CME | 1,2,3,5 |
| Furnes B et al [33] | 2018 | CME+CVL | 1,2 |
| Galizia G et al [34] | 2013 | CME+CVL | 1,2 |
| Gao Z et al [35] | 2018 | CME | 1,2 |
| Gaupset R et al [36] | 2018 | D3 | 1,2,3,4,5,6,7 |
| Gouvas N et al [37] | 2012 | CME | 1,2 |
| Hamzaoglu I et al [38] | 2018 | CME | 1,2,3,5,6 |
| Han DP et al [39] | 2013 | D3 | 1,2,3,5,8 |
| Han DP et al [40] | 2014 | D3 | 1,2,3,5,8 |
| He Z et al [41] | 2019 | CME | 1,2,3,5 |
| Ho MLL et al [42] | 2019 | CME+CVL | 1,2,3,5 |
| Hohenberger W et al [9] | 2009 | CME+CVL | 1,2,3,6,7,8 |
| Huang JL et al [43] | 2015 | CME | 1,2,3,5 |
| Kanemitsu Y et al [44] | 2013 | D3 | 1,3,4,5,8 |
| Karachun A et al [45] | 2019 | D3 | 1,3 |
| Kataoka K et al [46] | 2020 | D3 | 1,3 |
| Killeen s et al [47] | 2014 | CME | 1,2,3 |
| Killeen S et al [48] | 2014 | CME | 1,2,3,7,8 |
| Kim CW et al [49] | 2016 | mCME | 1,2,3,5,8 |
| Kim IY et al [50] | 2016 | mCME | 1,2,3,5,8 |
| Kim NK et al [51] | 2016 | CME | 1,2,3,5,8 |
| Kim JS et al [52] | 2021 | D3 | 1,2,3 |
| Kobayashi H et al [53] | 2020 |  | 1,2,3,7,8 |
| Koc MA et al [54] | 2021 | CME+CVL | 1,2,5,8 |
| Lan YT et al [55] | 2010 | D3 | 1 |
| Larach JT et al [56] | 2021 | CME+CVL | 1,2,3,5 |
| Lee SD et al [57] | 2009 | CME | 1,2,3 |
| Lee JM et al [58] | 2020 | D3 | 1,2,3,5,8 |
| Li J et al [59] | 2020 | CME | 1,2,3,5,8 |
| Liang JT et al [60] | 2015 | D3+CME | 1,2,3,5 |
| Livadaru C et al [61] | 2019 | CME+CVL | 1,2 |
| Luglio G et al [62] | 2015 | CME | 1,2 |
| Melich G et al [63] | 2014 | CME | 1,2,5 |
| Merkel S et al [64] | 2016 | CME | 1,2 |
| Mori S et al [65] | 2015 | CME | 1,2,3 |
| Mori S et al [66] | 2014 | CME+CVL | 1,2,3,5 |
| Nagasaki T et al [67] | 2015 | CME | 1,2 |
| Nakajima K et al [68] | 2014 | D3 | 1,3 |
| Olmi S et al [69] | 2020 | CME+CVL | 1,2,3,4,5,6 |
| Olofsson F et al [70] | 2016 | CME+CVL | 1 |
| Ouyang M et al [71] | 2019 | CME | 1,2,3,8 |
| Ow ZGW et al [72] | 2020 | CME | 1,2,3,7,8 |
| Ozben V et al [73] | 2018 | CME | 1,2,3,5,6,8 |
| Pedrazzani C et al [74] | 2018 | CME | 1,3 |
| Perrakis A et al [75] | 2018 | CME | 1,2,3,6,8 |
| Petz W et al [76] | 2017 | CME | 1,2,3,4 |
| Pramateftakis MG et al [77] | 2010 | CME | 1,2 |
| Prevost GA et al [78] | 2018 | CME+CVL | 1,2,3,8 |
| Ramachandra C et al [79] | 2020 | CME+CVL | 1,3,5,8 |
| Rinne JKA et al [80] | 2019 | CME | 1,3 |
| Sahara K et al [81] | 2020 | D3 | 1,2,3,5 |
| Sammour T et al [82] | 2019 | CME+CVL | 1,2 |
| Sheng QS et al [83] | 2017 | CME | 1,3,5,6,8 |
| Shin JW et al [84] | 2014 | mCME | 1,2 |
| Shin JK et al [85] | 2018 | D3+CME | 1,2,5 |
| Siani LM et al [86] | 2014 | CME+CVL | 1,2,3,4,5,6,8 |
| Siddiqi N et al [87] | 2020 | CME | 1,2,3,5,8 |
| Spinoglio G et al [88] | 2016 | mCME | 1,2,5,6 |
| Spinoglio G et al [89] | 2018 | CME | 1,2,5 |
| Storli KE et al [90] | 2013 | CME | 1,2,3 |
| Storli KE et al [91] | 2014 | CME | 1 |
| Subbiah R et al [92] | 2015 | CME+CVL | 1,7 |
| Takahashi H et al [93] | 2016 | CME | 1,2,8 |
| Takemasa I et al [94] | 2013 | CME+CVL | 1,2,8 |
| Thorsen Y et al [95] | 2016 | D3 | 1,3,4,5 |
| Thorsen Y et al [96] | 2019 | D3 | 1,3,4,5 |
| Tominaga T et al [97] | 2021 | mCME | 1,3 |
| Wang Y et al [98] | 2017 | CME+CVL | 1,2,3,7,8 |
| Wei M et al [99] | 2018 | D3+CME | 1,2,3,8 |
| West NP et al [100] | 2010 | CME | 1,2,3,4,5,6,8 |
| Willard CD et al [101] | 2018 | D3 | 1,3,4,5 |
| Wu QB et al [102] | 2016 | CME | 1,8 |
| Wu H et al [103] | 2020 | CME+CVL | 1,2,3,5,8 |
| Xie D et al [104] | 2016 | D3+CME | 1,2,3,5,8 |
| Yamamoto M et al [105] | 2019 | CME+CVL | 1,2 |
| Yan D et al [106] | 2020 | CME | 1,5,7 |
| Yang Y et al [107] | 2019 | CME+CVL | 1,2,3,8 |
| Yi X et al [108] | 2019 | D3 | 1,3,4,5 |
| Yozgatli TK et al [109] | 2019 | CME | 1,2,3,5,6,8 |
| Zedan A et al [110] | 2021 | CME | 1,3,8 |
| Zhao LY et al [111] | 2014 | D3 | 1,3,5,6 |
| Zhao LY et al [112] | 2014 | D3 | 1,6 |
| Zurleni T et al [113] | 2018 | CME | 1,2,3,6,7,8 |

RRC: radical right colectomy; CME: complete mesocolic excision; CVL: central vascular ligation; mCME: modified complete mesocolic excision
